# Flexion teardrop fracture of the cervical spine: a narrative review

**DOI:** 10.1530/EOR-2025-0010

**Published:** 2025-10-01

**Authors:** Ignacio Cirillo, Sebastián Blanco, Sebastián Cabello, Guillermo Ricciardi, Alfredo Guiroy, Ratko Yurac, on behalf AO Spine Latin America Trauma Study Group

**Affiliations:** ^1^ Hospital del Trabajador, Santiago, Chile; ^2^ Clínica Las Condes, Santiago, Chile; ^3^ Universidad Andrés Bello, Hospital del Trabajador, Facultad de Medicina, Santiago, Chile; ^4^ Orthopedic and Traumatology, Universidad de los Andes, Santiago, Chile; ^5^ Clínica Universidad de los Andes, Santiago, Chile; ^6^ Clínica RedSalud Santiago, Santiago, Chile; ^7^ Centro Médico Integral Fitz Roy, Buenos Aires, Argentina; ^8^ Sanatorio Güemes, Buenos Aires, Argentina; ^9^ Hospital General de Agudos Dr Teodoro Álvarez, Buenos Aires, Argentina; ^10^ Clínica de Cuyo, Mendoza, Argentina; ^11^ Elite Spine Health and Wellness Center, Port St. Lucie, Florida, USA; ^12^ Department of Orthopedic and Traumatology, University del Desarrollo, Santiago, Chile; ^13^ Spine Unit, Department of Traumatology, Clínica Alemana, Santiago, Chile

**Keywords:** cervical spine, teardrop fracture, flexion-compression injuries, trauma

## Abstract

- Teardrop fractures of the cervical spine are characterized by a triangular-shaped fragment located in the anteroinferior corner of the vertebral body.
- Flexion-type teardrop fractures are highly unstable injuries resulting from a flexion-compression mechanism.
- A notable feature of these injuries is retrolisthesis of the vertebral body, which is often associated with a high risk of neurological compromise.
- The anterior approach is the most commonly used surgical treatment for flexion-type teardrop fractures.
- In contrast, extension-type teardrop fractures primarily affect the axis vertebral body and are generally stable injuries that can be treated nonoperatively.

## Introduction

Spinal injuries frequently affect young males, with the subaxial cervical spine being one of the most affected segments. These injuries are typically associated with high-energy mechanisms and often occur in conjunction with other injuries. Car accidents are the leading cause of cervical fractures, accounting for up to 48% of cases, followed by falls (21%) and sports injuries (14%) (1). Polytrauma patients present with cervical spine injuries in up to 3.7% of cases, and the subaxial cervical spine is particularly vulnerable due to its mobility and proximity to the more rigid thoracic spine (2, 3, 4). This region accounts for 65% of cervical fractures and over 75% of dislocations across all spinal segments (1).

The teardrop fracture (TDF) is defined as a coronal fracture that affects the anterior inferior angle of the vertebral body (VB) (5). TDFs in the subaxial cervical spine are particularly severe due to their strong association with significant neurological compromise and the morbidity resulting from related injuries and neurological damage. Compared to other subaxial cervical spine fractures, teardrop fractures are less frequently reported and lack robust evidence to guide standardized treatment, resulting in greater controversy regarding their management (5, 6, 7).

We aim to provide a comprehensive overview of the existing literature on the diagnosis, management, and treatment of teardrop fractures in the cervical spine through a narrative review.

## Materials and methods

This narrative review of the literature was conducted using various scientific databases (PubMed, Cochrane Database, and Google Scholar) to gather current evidence on the biomechanical mechanisms, classifications, and diagnostic and treatment options for flexion teardrop fractures of the cervical spine. The literature search process followed the principles of systematic review, as outlined in the PRISMA 2020 statement. Although a structured literature search was performed, we opted for a narrative review format, which allowed us to summarize current knowledge and clinical experience while highlighting key controversies and areas for future research. This decision was based on the limited number of high-quality studies specifically addressing flexion teardrop fractures, as well as the significant heterogeneity in study design, patient populations, treatment strategies, and outcome measures.

Studies were selected according to the following criteria: experimental and observational studies published from any date to August 2024 that addressed the characteristics, diagnosis, and treatment of this specific injury, and were published in indexed journals with full-text availability. Due to the limited evidence available, descriptive studies were also included.

Our literature search strategy was based on using medical subject headings (MeSH) and entry terms related to teardrop fractures. The following search strategy was initially developed for the MEDLINE search: (‘flexion teardrop fracture’ OR teardrop [tiab] OR tear-drop [tiab] OR tear drop [tiab]) AND (spine [mesh] OR spinal [tiab] OR vertebral [tiab] OR vertebra [tiab] OR cervical [tiab]). The search was not restricted by study design or language; however, due to resource limitations, only studies published in English, Portuguese, or Spanish were ultimately included. The MEDLINE search strategy was initially used and then adapted to the syntax and subject headings of the other databases.

The selection process was conducted by four reviewers, divided into two groups, who independently screened the titles and abstracts according to the inclusion criteria. Disagreements were initially resolved by discussion among the reviewers and finally by a third opinion from an ‘expert’ researcher who was not involved as a reviewer in the screening and selection process.

After identifying relevant studies, the reviewers conducted a narrative review to synthesize information on surgical indications and therapeutic strategies for this injury. Our qualitative synthesis was organized into several sections, including definition, injury mechanism and biomechanical aspects, imaging and differential diagnoses, classification, and treatment.

## Results

In our research strategy, we initially identified 115 titles. After reviewing these titles and their abstracts, we selected 18 articles specifically related to our topic based on established criteria (5, 6, 7, 8, 9, 10, 11, 12, 13, 14, 15, 16, 17, 18, 19, 20, 21, 22, 23). We also included two articles discovered through manual citation searches to complete our narrative review (24, 25). The characteristics of the included articles are outlined in Table 1.

**Table 1:** Characteristics of included studies.

Study	Study design	TDF type	Participants (*n*)	Note
Kahn & Schneider (5)	RC	FT	NR	First FT TDF description
Torg *et al.* (6)	RC	FT	55	National Football Head and Neck Injury Registry
Kim *et al.* (7)	RC	Both	FT: 21; ET: 4	
Kim *et al.* (8)	RC	FT	45	
Kim *et al.* (9)	RC	ET	33	Clear indications for surgical cases
Basil & Kumar (10)	TN	FT	1	Anterior fixation without corpectomy
Fisher *et al.* (11)	RC	FT	45	Comparison of surgery versus halo vest
Haupt *et al.* (12)	RC	FT	5	
Hu *et al.* (13)	RC	ET	16	Comparison of non-operative versus surgical management
Isla *et al.* (14)	RC	FT	30	
Johnson & Cannon (15)	RC	FT	10	All were treated non-operatively
Mori *et al.* (16)	RC	FT	5	One of the first cohorts with CT imaging
Korres *et al.* (17)	RC	FT	38	
Korres *et al.* (18)	RC	ET	14	
Cabanela & Ebersold (19)	RC	FT	8	
Ianuzzi *et al.* (20)	CS	FT	NA	Compared different types of fixations
Signoret *et al.* (21)	RC	FT	8	Only-posterior surgical approach
Wang *et al.* (22)	RC	ET	15	Anterior fusion for extension-type fractures
Watanabe *et al.* (23)	RC	ET	13	
Korres *et al.* (24)	RC	FT	54	
Boran *et al.* (25)	RC	ET	7	

RC, retrospective cohort; CS, cadaveric study; TN, technical note; NR, not reported; CT, computed tomography; FT, flexion type; ET, extension type.

### Definition and clinical features

In the context of cervical fractures, the term ‘teardrop’ refers to an injury pattern characterized by a triangular anteroinferior bone fragment of the VB (5, 6). This injury can be associated with two distinct clinical scenarios, each differing in injury mechanisms, mechanical instability, treatment approaches, and prognosis. These scenarios include flexion-type TDF fractures and extension-type TDF fractures (5, 6, 7).

**Figure 1:**
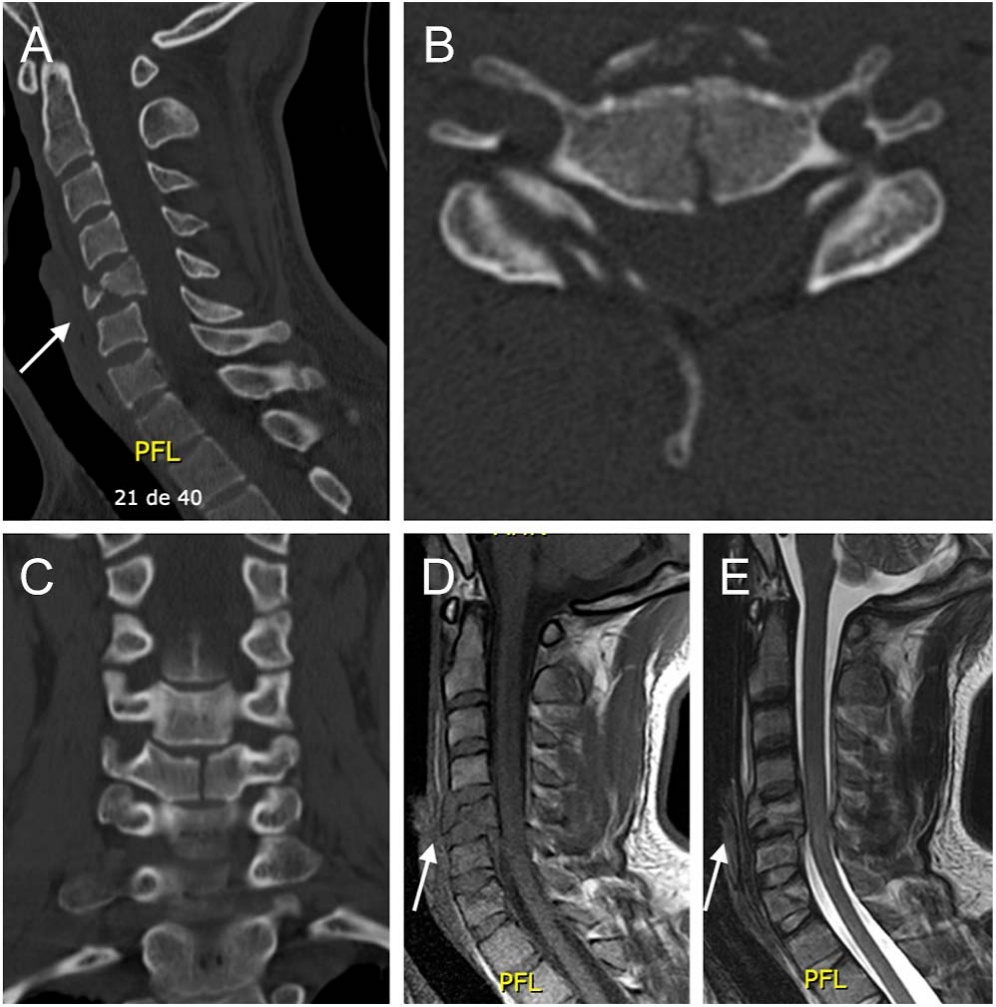
Sagittal (A), axial (B), and coronal (C) CT scans showing a C5 teardrop fracture. Sagittal reconstruction using MRI in T1 (D) and T2 (E) sequences of the same lesion, showing spinal cord signal changes and posterior disc and PLL disruption.

**Figure 2:**
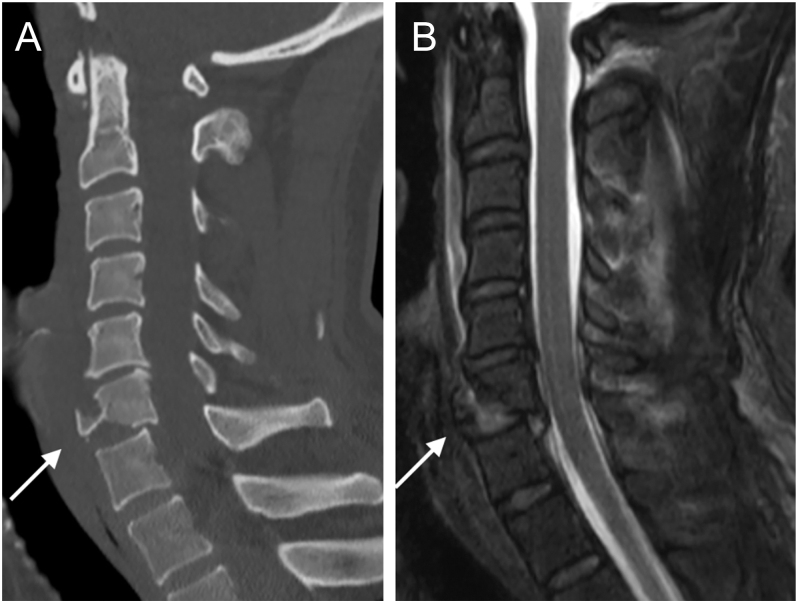
CT and MRI study of a C6 teardrop fracture. An anteroinferior fragment is observed, connected to the caudal VB by the disc and ALL (arrow). Notice the retrolisthesis of the larger C6 VB fragment, disc disruption, and spinal cord signal changes.

Schneider and Kahn described the acute flexion-type TDF fracture, which is characterized by the separation and downward and forward displacement of the anterior inferior margin of the affected VB (5). Simultaneously, the posterior inferior margin of the same VB is displaced posteriorly into the spinal canal. Acute flexion-type TDFs are associated with the complete rupture of both the anterior and posterior ligamentous structures, as well as the intervertebral disc, resulting in a high level of instability (Fig. 1). This injury pattern predominantly affects the subaxial cervical spine, particularly the C5 VB, in up to 74% of cases, followed by C4 (16%) and C6 (10%) (7, 14, 17, 18, 24). TDFs account for 8–23% of fractures resulting from a flexion-compression mechanism (24). Neurological compromise due to the retropulsion of the posteroinferior fragment can lead to devastating outcomes, resulting in a reduced spinal canal diameter and neurological impairment in over 50% of cases among large reported cohorts (Fig. 2) (7, 14, 17, 18, 24). Some authors have described different subtypes of flexion-TDF and examined their relationship with the risk of neurological involvement, which will be discussed in the classification section (6, 24).

Extension-type TDFs are a specific type of injury that differs from those previously mentioned (9, 18, 20, 22, 23). These fractures primarily involve the axis and are characterized by a triangular fragment of bone that is avulsed from the anteroinferior corner of the VB. This is an avulsion fracture caused by the intact anterior longitudinal ligament during hyperextension of the head and upper cervical spine (9, 18, 20, 22, 23), representing from 3 to 13% of cervical spine trauma (9, 18, 20, 22, 23). This type of injury is generally considered mechanically stable and usually does not lead to neurological deficits (18). Most patients with an extension-type TDF of the axis can be treated using conservative methods (18, 20). However, some authors have expressed concerns regarding the mechanical stability of this fracture (9, 22, 23).

### Injury mechanism and biomechanics

**Figure 3:**
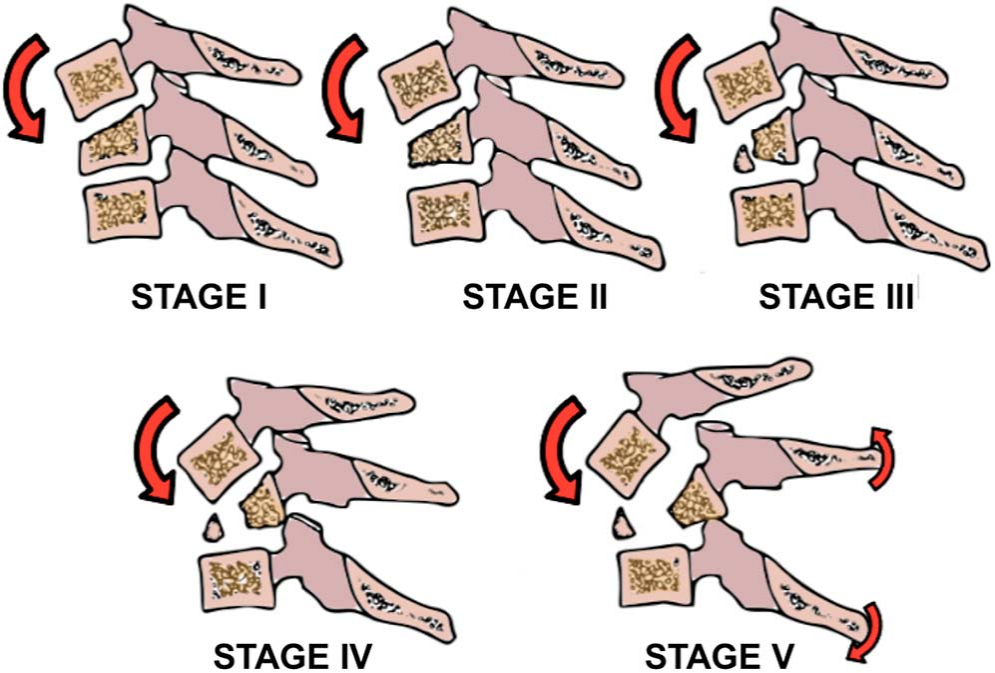
Diagram representing Allen *et al*.'s (26) classification.

It is important to differentiate the injury mechanisms of flexion and extension types of TDFs. For flexion-type TDFs of the subaxial cervical spine, the classic concepts presented by Allen *et al*. regarding injury progression should be considered (26). In 1982, Allen and Ferguson described eight types of deforming forces, each associated with characteristic injury patterns that help link imaging findings to specific mechanisms of injury. Understanding the flexion-compression mechanism is essential for this review. This mechanism involves axial compressive loading combined with simultaneous flexion of the anteroinferior portion of the VB, with the center of rotation located at the anterior part of the VB, resulting in compression of the anterior column. The injury progresses through several stages. The first stage involves wedge compression of the VB. In the second stage, there is a greater loss of vertebral height, leading to localized kyphosis (also known as beaking of the VB). At this point, the posterior longitudinal ligament (PLC) remains intact, and there is no retropulsion into the spinal canal. As the energy involved increases, a coronal fracture develops, creating an anterior triangular fragment that separates from the VB. At this stage, disruption of the PLC may occur or may not. Stage four is characterized by retrolisthesis of the VB, measuring less than 3 mm, while significant PLC and intervertebral disc disruption, angular kyphosis, the presence of an anteroinferior fragment, and considerable retrolisthesis mark stage five. This progression describes the development of a flexion-type TDF (Fig. 3). This type of injury is commonly observed in car accidents, specific sports injuries, and diving into shallow water.

TDFs of the axis occur as a result of hyperextension, which leads to compression of the posterior bony structures that act as a fulcrum (18). This type of injury results in an avulsion fracture at the lower anterior corner of the VB due to stretching of the anterior longitudinal ligament. The fracture line starts at the front of the VB and extends backward and downward. In addition, this injury can lead to a rupture of the intervertebral disc at the C2–3 level. If hyperextension continues, the damage may extend to the posterior ligamentous structures, potentially causing posterior displacement of the C2 VB (18).

Extension-type TDFs occur in various trauma scenarios, including both high-energy trauma and low-energy trauma in elderly patients (9, 18, 20, 22, 23, 24, 25).

### Imaging

Imaging studies play an essential role, with plain radiography and especially computed tomography (CT) providing valuable diagnostic and characterization information to guide both initial and definitive management. Cervical spine plain radiographs provide information on cervical spine alignment, potential prevertebral soft tissue swelling, and fracture characteristics, including the anteroinferior triangular fragment and posterior retropulsed VB fragment.

Dynamic cervical radiographs taken in flexion and extension have been described by Korres *et al.* as a means to identify hidden ligamentous instability, especially in cases of non-displaced fractures (24). However, due to the potential neurological risks associated with flexion-extension maneuvers in cases of highly unstable lesions, the authors of this review express concerns about routinely performing these techniques.

CT is now highly available in specialized emergency services, and it is critical for the specific characterization of the oblique coronal fracture lines that define this teardrop fragment and the sagittal fracture line of the posterior VB fragment (16). In addition, CT can visualize possible retropulsion of the posterior VB fragment, as well as indirect signs of PLC injury, such as increased interspinous distance and VB dislocation (7, 14, 17, 18, 24). Kim *et al.* described several factors that can predict nonunion or failure of nonoperative management in the assessment of extension avulsion fractures of the axis (9). Assessment of CT imaging should consider the following: an avulsion fracture ratio greater than 43%, a fracture displacement exceeding 5 mm, or the presence of associated injuries to C2. These factors can increase the risk of nonunion in conservatively managed anterior C2 teardrop fractures (9).

Angio-CT also plays a role in this fracture due to the cervical anatomy and its proximity to critical vascular structures, such as the vertebral artery, which runs between C6 and C1 through the transverse foramina. When suspected, this imaging modality should be requested (27).

### Classifications

**Figure 4:**
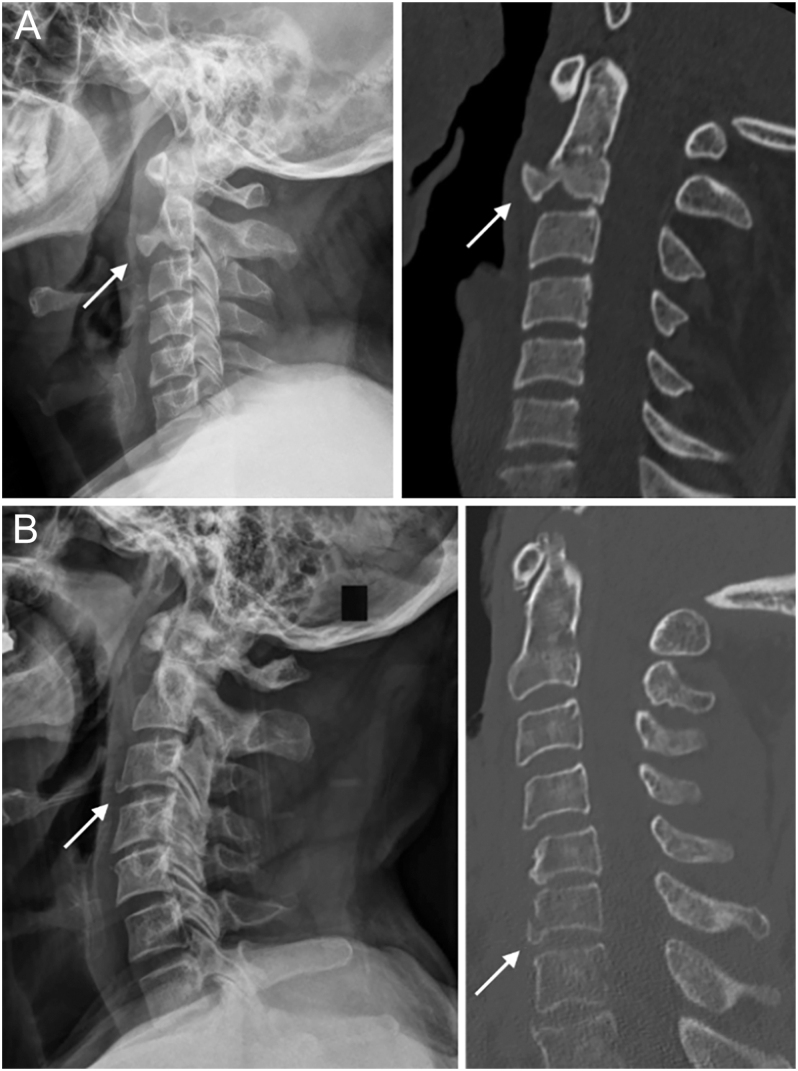
(A and B) Extension teardrop fracture of C2. Left: X-ray, right: CT. (C and D) Subaxial extension teardrop fracture. Left: X-ray, right: CT.

The AOSpine subaxial cervical spine injury classification system is widely used by spinal surgeons worldwide (28). Flexion-type TDFs of the subaxial cervical spine are classified as type C because they display clear evidence of translation, such as the retropulsed vertebra. These fractures may also involve distraction of both the anterior and posterior vertebral elements, categorizing them as translational injuries. In contrast, extension-type TDFs are typically classified as type A1 since they affect only one VB endplate (Fig. 4). However, to accurately determine the type of injury and its mechanical stability, a comprehensive assessment of both the anterior and posterior elements is essential.

Korres *et al.* developed a classification system for flexion-type TDFs based on the morphology of these injuries, utilizing radiographs, CT scans, and MRI (17, 24). In their retrospective analysis of 54 cases, they correlated four different types with mechanical stability, neurological impairment, and treatment decision-making (24).

Type I corresponds to a minor fracture of the anteroinferior VB angle, measuring less than 3 mm, without a sagittal fracture of the posterior half of the VB or retrolisthesis. Type II is characterized by a coronal fracture of the anteroinferior VB corner larger than 3 mm, accompanied by a sagittal fracture of the posterior half of the VB, but without retrolisthesis. They reported a low association of neurological compromise according to the ASIA scale, which ranged from 19% in type I to 28.5% in type II.

Type III is further divided into two subtypes (a and b) based on the degree of retrolisthesis. If the displacement is less than 4 mm, the lesion is classified as type IIIa; if it exceeds 4 mm, it is classified as type IIIb. Type IV is characterized, apart from the previously described fractures, by the presence of a locked facet and anterior dislocation of the above vertebra. The authors concluded that types IIIa, IIIb, and IV have an absolute indication for operative treatment, usually by an anterior or posterior approach.

**Figure 5:**
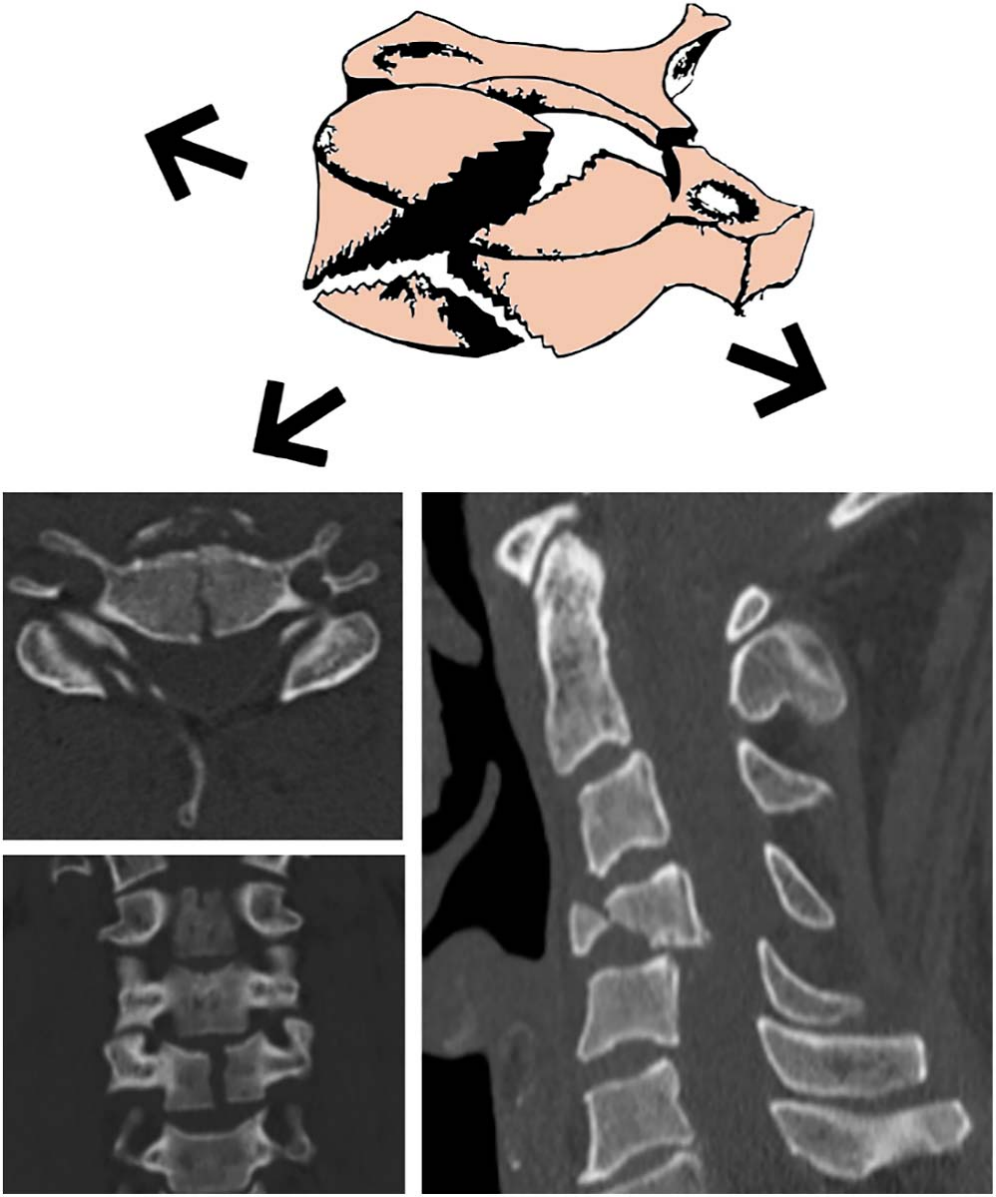
Diagram representing a three-part, two-plane flexion teardrop fracture. Below are CT images illustrating the three planes.

Torg *et al.* describe two flexion-type TDFs. The first is the simple type, characterized by an isolated coronal fracture with a low association with neurological involvement (6). Type II is a three-part fracture in two planes, where the coronal fracture divides the VB into an anterior and a posterior fragment, the latter presenting with a sagittal fracture line. This type is highly associated with neurological impairment and should be suspected and specifically looked for in CT scans (Fig. 5).

### Definitive treatment and experience

The inherent instability of flexion-type TDFs explains the general preference for surgical management in most cases, which often leads to more favorable outcomes (5, 6, 7, 8, 10, 11, 14, 17, 20, 21, 24). Reports indicate that conservative management can result in poor outcomes, including late kyphosis in up to 100% of cases, neurological deficits in 20%, delayed surgery, and nonunion (20). According to Korres *et al.*, only type I fractures are effectively managed with conservative treatment. Type II fractures may be candidates for conservative treatment if there are no neurological symptoms or signs of instability evident in CT and MRI scans (25).

**Figure 6:**
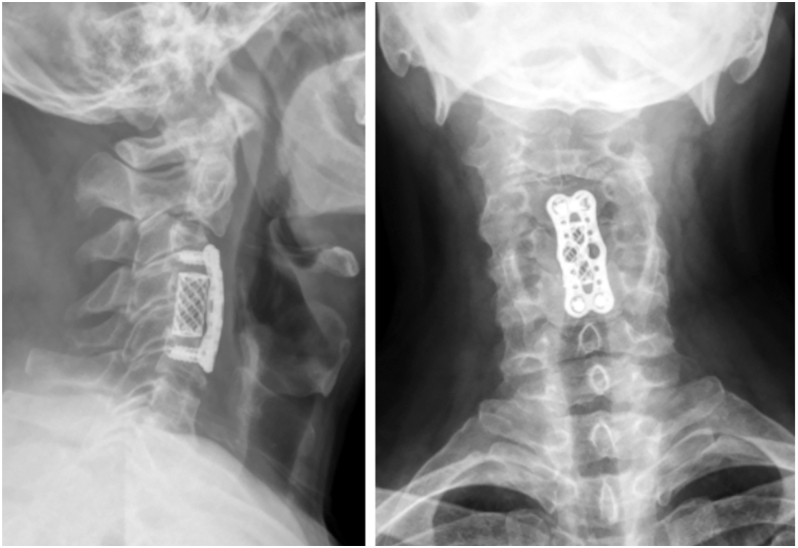
Resolution of a teardrop fracture using an anterior approach with a mesh-type cage and anterior plate fixation.

**Figure 7:**
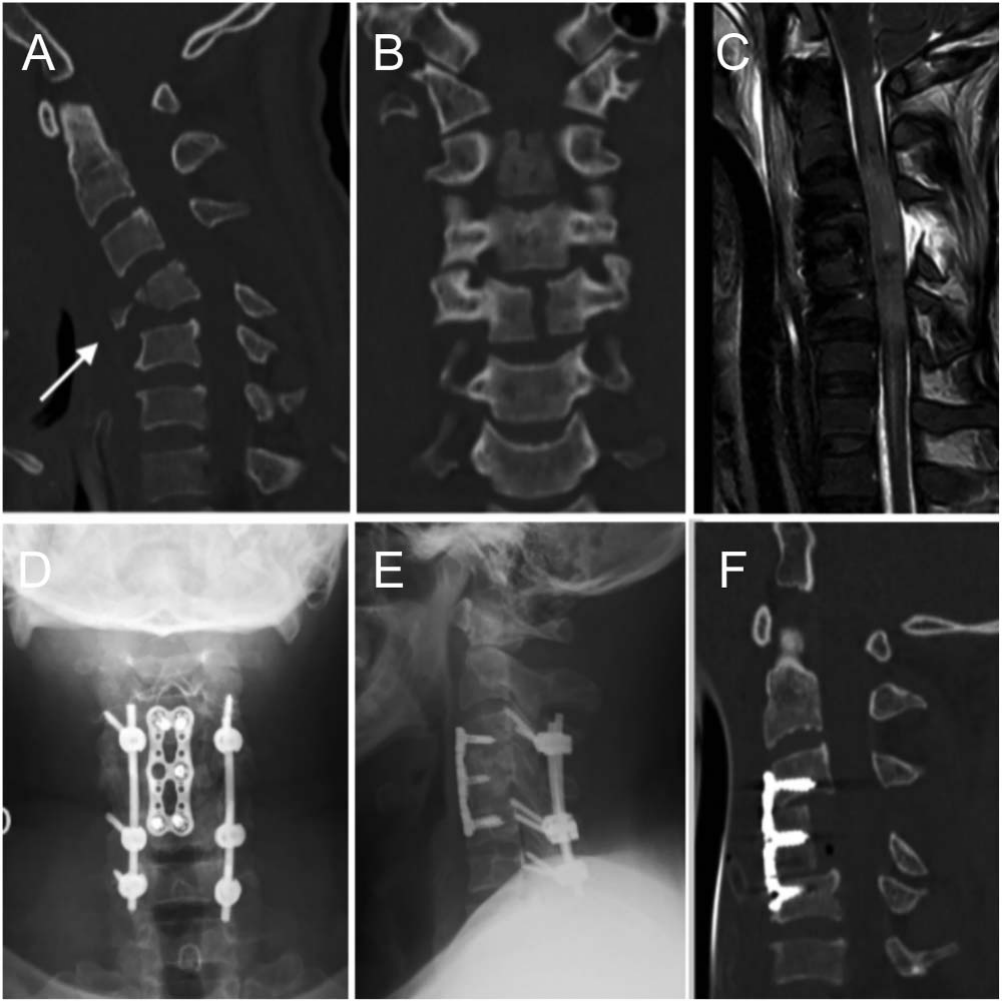
C3 teardrop fracture. (A and B) Sagittal and coronal CT scans of the spine. C: MRI showing signal alterations. (D and E) Postoperative X-rays after combined anterior and posterior fixation, plus autograft. (F) Postoperative CT scan.

**Figure 8:**
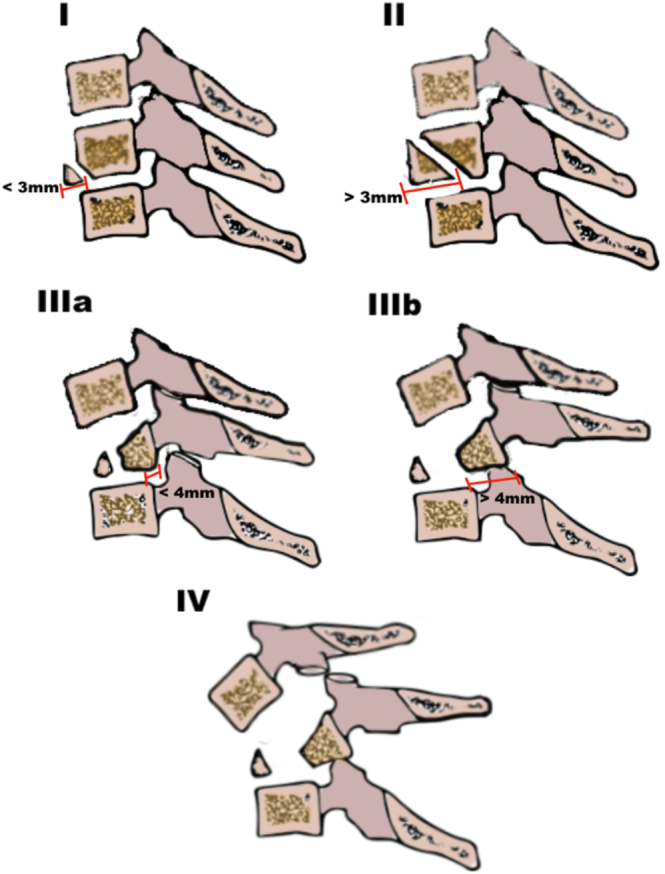
Diagram representing Korres *et al.*’s (24) classification.

Although data guiding treatment decisions for flexion-type TDFs are limited to small retrospective cohorts, a consistent trend favors the anterior surgical approach (7, 11, 12, 14, 19). Anterior corpectomy and plate stabilization were remarked as an effective treatment for the flexion-type teardrop fracture (7, 11, 12, 14, 19). Fisher *et al.* indicate that anterior cervical plating is a safe and effective treatment for cervical teardrop fractures and that it is superior to the halo thoracic vest for restoring and maintaining sagittal alignment and for minimizing treatment failures (11). Reconstruction of the anterior column is carried out using mesh-type cages, expandable cages, or structural bone grafts (either autologous or donor) (Fig. 6) (Fig. 7) (7, 11, 12, 14, 19). As an alternative to corpectomy, Basil *et al.* proposed open reduction and internal fixation of a cervical teardrop fracture in patients with an anteriorly displaced fragment and without neurologic deficit (14). The use of this strategy, avoiding corpectomy, could potentially offer several significant advantages, such as shorter operative time, less blood loss, reduced risk of pseudoarthrosis, lower risk of esophageal or neural injury, reduced implant and graft-associated costs, and decreased complications at the donor site (Fig. 8).

To explore alternatives to the anterior approach, it is essential to highlight the cadaveric study by Iannuzzi *et al.*, which compared various surgical constructs in anatomical specimens to assess whether the type of fixation (anterior, posterior, or combined) affected the stability of a cervical flexion TDF at the C5–C6 level (20). They found that all constructs restored stability comparable to that of intact specimens. In line with these findings, Signoret *et al.* reported that reducing cervical flexion teardrop fractures via a posterior approach in neurologically intact patients may offer advantages in terms of simplicity, as the fracture can be reduced through a single approach without bone excision or grafting, and appears to be cost-effective (21). Korres *et al.* reported that 17 of 54 patients with flexion-type TDF underwent surgical treatment, predominantly via an anterior approach; however, five patients were managed with a posterior approach and also achieved favorable outcomes. The authors concluded that surgical stabilization may be equally effective, regardless of whether it is performed through an anterior, posterior, or combined approach (24).

For extension-type TDFs, conservative treatment with a hard cervical collar is generally the rule, as reported in most cohorts (9, 13, 15, 18, 22, 23, 25). However, surgical stabilization may be indicated in specific situations. Reasonable indications include an avulsion fragment involving more than 43% of the VB height, fracture displacement greater than 5 mm, associated injuries to C2, intervertebral disc injury, neurological deficit, or radiographic signs of instability (9, 13).

## Discussion

As noted in our narrative review, so-called TDFs involve two distinct types of injuries, each with a different prognosis and treatment approach. First, highly unstable flexion-type TDFs often require anterior surgical stabilization. On the other hand, extension-type TDFs typically affect the anteroinferior corner of the axis, and, with a fair description among low-energy trauma in elderly patients, are predominantly treated through nonoperative management with few exceptions (5, 6, 7, 8, 9, 10, 11, 12, 13, 14, 15, 16, 17, 18, 19, 20, 21, 22, 23, 24, 25).

Although the first descriptions of these injury patterns date back more than 70 years, these fractures remain rarely reported in the literature (5). The largest subaxial cohorts consist of only small case series, typically with fewer than 15 patients, often combined with other fracture types. As a result, decision-making is supported by very low-quality evidence. To date, the largest reported cohort of flexion-type teardrop fractures is that of Korres *et al.*, which included 54 patients (24). The largest extension-type cohort was reported by Kim *et al.*, comprising 33 patients (9).

According to the surgical approach to subaxial cervical spinal injuries, the literature has shown that fractures involving all three columns benefit from anterior stability, which is superior to posterior fixation alone, and alignment is more adequately restored (29). However, adding posterior instrumentation can add even greater stability (30, 31, 32, 33). The anterior approach, however, has advantages in terms of reduced intraoperative bleeding and postoperative pain (29, 30, 31, 32). Flexion-type TDFs adhere to this statement, and the anterior approach should be the standard (7, 11, 12, 14, 19). On the other hand, the posterior approach could be elected in certain situations, such as neurologically intact patients (21, 24).

Our study has limitations. As a narrative review, it is susceptible to potential selection bias, as the studies selected for inclusion were chosen based on the authors’ discretion. Furthermore, the evidence supporting this narrative review is primarily comprised of retrospective observational studies, which are prone to a high risk of bias and limit the generalizability of the conclusions drawn. However, this narrative review offers a comprehensive overview of subaxial cervical teardrop fractures, facilitating a deeper understanding of the topic.

Future studies, preferably prospective and randomized controlled trials, are needed to compare the outcomes of different surgical approaches, as well as the effectiveness of conservative management, and to validate or develop new classification systems.

## Conclusion

Flexion teardrop fractures are highly unstable cervical spine injuries resulting from a flexion-compression mechanism. Retrolisthesis of the VB is a hallmark feature and is frequently associated with significant neurological compromise. Management depends on the specific characteristics and stability of the fracture, although surgical intervention is typically required. The anterior approach is most commonly used, while combined anterior-posterior or posterior-only approaches are reserved for selected cases. In contrast, extension-type teardrop fractures, most widely seen at C2, tend to be stable and are often managed conservatively unless specific radiological or clinical features suggest instability.

## ICMJE Statement of Interest

There is no conflict of interest that could be perceived as prejudicing the impartiality of the research reported.

## Funding Statement

This research did not receive any specific grant from any funding agency in the public, commercial, or not-for-profit sector.
